# Addressing Challenges When Applying GRADE to Public Health Guidelines: A Scoping Review Protocol and Pilot Analysis

**DOI:** 10.3390/ijerph19020992

**Published:** 2022-01-16

**Authors:** Lucia Kantorová, Tereza Friessová, Simona Slezáková, Alena Langaufová, Jiří Kantor, Zachary Munn, Timothy Hugh Barker, Srinivasa Vittal Katikireddi, Reem A. Mustafa, Marija Franka Žuljević, Marina Lukežić, Jitka Klugarová, Abanoub Riad, Tereza Vrbová, Andrea Pokorná, Petra Búřilová, Jiří Búřil, Aleksandar Kirkovski, Nensi Ćaćić, Ljerka Delač, Ružica Tokalić, Tina Poklepović Peričić, Miloslav Klugar

**Affiliations:** 1 Czech National Centre for Evidence-Based Healthcare and Knowledge Translation (Cochrane Czech Republic, Czech EBHC: JBI Centre of Excellence, Masaryk University GRADE Centre), Institute of Biostatistics and Analyses, Faculty of Medicine, Masaryk University, Kamenice 753/5, 625 00 Brno, Czech Republic; lucia.kantorova@mail.muni.cz (L.K.); tereza.friessova@med.muni.cz (T.F.); simona.slezakova@med.muni.cz (S.S.); alena.langaufova@med.muni.cz (A.L.); klugarova@med.muni.cz (J.K.); abanoub.riad@med.muni.cz (A.R.); tereza.vrbova@med.muni.cz (T.V.); apokorna@med.muni.cz (A.P.); burilova@med.muni.cz (P.B.); jirka210312@gmail.com (J.B.); 2 Department of Public Health, Faculty of Medicine, Masaryk University, Kamenice 753/5, 625 00 Brno, Czech Republic; 3 Center of Evidence-Based Education & Arts Therapies: A JBI Affiliated Group, Faculty of Education, Palacky University, Žižkovo nám. 5, 779 00 Olomouc, Czech Republic; jiri.kantor@upol.cz; 4 Institute of Special Education Studies, Faculty of Education, Palacky University, Žižkovo nám. 5, 779 00 Olomouc, Czech Republic; 5 JBI, Faculty of Health and Medical Sciences, The University of Adelaide, Adelaide, SA 5005, Australia; zachary.munn@adelaide.edu.au (Z.M.); timothy.barker@adelaide.edu.au (T.H.B.); 6 MRC/CSO Social & Public Health Sciences Unit, University of Glasgow, Berkeley Square, 99 Berkeley Street, Glasgow G3 7HR, UK; vittal.katikireddi@glasgow.ac.uk; 7 Department of Health Research Methods, Evidence, and Impact, McMaster University, 1280 Main Street West, Hamilton, ON L8S 3L8, Canada; rmustafa@kumc.edu; 8 Department of Internal Medicine, University of Kansas Medical Center, 3901 Rainbow Boulevard, Kansas City, KS 66160, USA; 9 Department of Medical Humanities, School of Medicine, University of Split, Šoltanska 2, 21000 Split, Croatia; marija.franka.zuljevic@mefst.hr; 10 Department of Public Health, School of Medicine, University of Split, Šoltanska 2, 21000 Split, Croatia; mlukezic@outlook.de; 11 Department of Health Sciences, Faculty of Medicine, Masaryk University, Kamenice 753/5, 625 00 Brno, Czech Republic; 12 Ist Department of Neurology, St. Anne´s Faculty Hospital, Faculty of Medicine, Masaryk University, Pekařská 664/53, 656 91 Brno, Czech Republic; 13 PZU MK & RR Centar Medikal, 7000 Bitola, Macedonia; kirkovskialek@gmail.com; 14 Department of Research in Biomedicine and Health, School of Medicine, University of Split, Šoltanska 2, 21000 Split, Croatia; nensi.cacic@mefst.hr (N.Ć.); rtokalic@gmail.com (R.T.); tinapoklepovic@gmail.com (T.P.P.); 15 Department of Basic and Clinical Pharmacology and Toxicology, Faculty of Medicine, University of Rijeka, Braće Branchetta 20, 51000 Rijeka, Croatia; ljerka.delac@medri.uniri.hr

**Keywords:** GRADE, guidelines, public health, methodology, challenges, scoping review protocol

## Abstract

This is a protocol for a scoping review that aims to determine how guideline authors using the Grading of Recommendations, Assessment, Development, and Evaluations (GRADE) approach have addressed previously identified challenges related to public health. The Joanna Briggs Institute (JBI) methodology for scoping reviews will be followed. We will search and screen titles of guidelines for all languages published in 2013–2021 in: the GIN library, BIGG database, Epistemonikos GRADE guidelines repository, GRADEpro Database, MAGICapp, NICE and WHO websites. Two reviewers will independently screen full texts of the documents identified. The following information will be extracted: methods used for identifying different stakeholders and incorporating their perspectives; methods for identification and prioritization of non-health outcomes; methods for determining thresholds for decision-making; methods for incorporating and grading evidence from non-randomized studies; methods for addressing concerns with conditional recommendations in public health; methods for reaching consensus; additional methodological concerns; and any modifications made to GRADE. A combination of directed content analysis and descriptive statistics will be used for data analysis, and the findings presented narratively in a tabular and graphical form. In this protocol, we present the pilot results from 13 identified eligible guidelines issued between January and August 2021. We will publish the full review results when they become available.

## 1. Introduction

Guidelines have been defined as ‘systematically developed evidence-based statements which assist providers, recipients and other stakeholders to make informed decisions about appropriate health interventions’ [1]. As such, guidelines require a rigorous and transparent approach for development, and developers should aim to meet standards in their development by groups such as the Guidelines International Network (GIN) [2]. Guideline developers across the globe are increasingly endorsing the Grading of Recommendations, Assessment, Development, and Evaluations (GRADE) approach for guideline development, a trustworthy and sensible approach for moving from the evidence to making a recommendation [3]. The GRADE’s output are evidence summaries (with assessment of the certainty of the evidence) and graded recommendations (with assessment of the strength of a recommendation and an overall certainty of the evidence). It is being used for all types of evidence synthesis and for the development of guidelines [3].

Guidelines may be developed across many different fields, including clinical practice, environmental health, and public health. Public health guidelines may be challenging to develop, partly due to the complex nature of the interventions assessed in such guidelines [4]. In public health, policy makers go beyond the usual efficacy and safety aspects of interventions, as is more common in clinical practice guidelines, and guidance on how to deliver interventions is just as important [4]. What constitutes a “public health guideline” has not been exactly defined, and although some organizations use the term to define their guidelines, most do not label the type of developed guidance as clearly. The most important aspect in defining a public health guideline seems to be the population perspective (rather than the perspective of the individual), the complexity of the interventions, and the scope encompassing broader policies, health reforms, population-wide interventions, with the respective target users (such as policy makers, governments, community leaders, relevant organizations). 

The GRADE Public Health Group (the Group) was approved by the GRADE Guidance Group in October 2017 to improve the methodology of applying GRADE in the development of public health guidelines [5]. The Group conducted a scoping review investigating the experiences of applying GRADE in public health and existing research activity in this area [5]. The result was an overview of current scientific knowledge in the field and the challenges identified in the literature and by experts, which include difficulties incorporating diverse perspectives in guideline panels, selecting outcomes (especially non-health outcomes), interpreting outcomes and identifying a threshold for decision-making, assessing the certainty of evidence from diverse sources, and addressing implications for decision-makers (e.g., concerns about conditional recommendations, or strong ones based on very low certainty of evidence) [5].

The Group proposed the following solutions to answer the identified challenges: to identify the training needs of public health guideline developers (and their stakeholders) in understanding and using the GRADE concept; to develop and disseminate detailed examples of the application of GRADE to public health topics; to adapt GRADE training materials for public health and public policy audiences [5]. This paper mainly addresses the second proposed solution and aims to provide both concrete examples of how GRADE is currently being used in public health guidelines and a discussion on which parts of the current GRADE guidance need to be adapted to public health topics.

In the proposed scoping review, we intend to build on the work of the Group by searching for GRADE public health guidelines and determining how the guideline authors may (or may not) have approached the challenges identified by the recent paper, if encountered within the guideline development process, and document any additional challenges or modifications to GRADE. We aim to analyze the guideline authors´ experience, methods, and examples related to these specific challenges, as described in the guidelines themselves.

A preliminary search of MEDLINE, Epistemonikos, PROSPERO, and Open Science Framework (OSF) was conducted on 22 August 2021, and no current or ongoing systematic reviews or scoping reviews on the topic were identified.

The aim of this review will be to identify the key methodological characteristics of public health guidelines that used the GRADE approach, particularly in terms of how developers addressed or overcame challenges when applying GRADE. We chose a scoping review as the appropriate method as it aligns with the purpose of the review to provide an overview of the methods used by guideline authors in relation to the observed challenges when applying GRADE to public health guidelines [6]. The review does not aim to assess the guideline methodological quality or develop methodological guidance on addressing possible challenges, nor will it be an overview of recommendations.

The results of the review will be used to further address the challenges of using GRADE in the development of public health guidelines, and, together with other research, will form a basis for GRADE concept articles or guidance on the topic. The work will benefit all relevant stakeholders (public health policy makers, governmental and non-governmental organizations, professional organizations, and all end-users of public health interventions) involved in the development, dissemination, and implementation of public health guidelines by allowing for a production of more trustworthy guidelines in public health. Too often, public health interventions are being implemented that do not benefit the public fully or are not based on robust scientific evidence. This work aims to improve the methods and approaches to developing public health guidance so that activities for health protection are based on trusted and robust evidence, cost-effective (avoiding waste of resources), useful, acceptable to all relevant stakeholders including the public, feasible, and ethical. We aim to enhance the health and financial literacy of the wider lay public. The results of the full review will help overcome some of the commonly observed challenges when formulating public health recommendations, such as the complexity of interventions and interpretations or situations when recommendations need to be formulated in the absence of robust evidence.

## 2. Methods

The proposed scoping review will be conducted in accordance with the Joanna Briggs Institute (JBI) methodology for scoping reviews [7,8] and will be informed mostly by the previous work of the GRADE Public Health group [5]. The Preferred Reporting Items for Systematic Reviews and Meta-analyses extension for scoping reviews (PRISMA-ScR [9]) will be used, bearing in mind the recently updated PRISMA 2020 [10] with changes relevant to scoping reviews described in the chapter on scoping reviews in the JBI manual on evidence synthesis [7].

According to the JBI methodology for scoping reviews, as with all good quality systematic reviews, an a priori protocol needs to be developed and published before undertaking the scoping review. The aim of such a protocol is to predefine the objectives, the review question and eligibility criteria, the detailed methods, and the reporting of the review, allowing for transparency of process. The protocol serves as a plan for the scoping review and should limit reporting bias. Any deviations of the scoping review from the protocol will be clearly highlighted and explained in the full scoping review [7].

An international team of experts in the field of guideline methodology and public health was assembled to cooperate on this scoping review. A piloting phase was conducted to improve the previously drafted methods and to further develop the necessary tools (e.g., for data extraction). After the piloting phase, the objectives have been rephrased more clearly, the eligibility criteria refined, the simple screening tool tested (see Appendix), and the data extraction tool modified and placed in an online form. In this section, we describe the basic methods of the proposed scoping review, followed by a presentation of the results of the piloting phase.

### 2.1. Review Question

How did guideline developers handle the specific challenges of applying the GRADE approach to developing recommendations in public health?

Challenges include: incorporating diverse perspectives in guideline panels, selecting and prioritizing outcomes (especially non-health outcomes), interpreting outcomes and identifying a threshold for decision-making, assessing the certainty of evidence from diverse sources, and addressing implications for decision-makers (e.g., concerns about conditional recommendations, or strong ones based on very low certainty of the evidence) [5].

We will aim to answer the following specific review questions:

1. How were previously identified challenges addressed within public health guidelines?
2. What additional challenges have been identified within public health guidelines?
3. Have any modifications been made to the GRADE approach within public health guidelines, and how were they justified?

### 2.2. Eligibility Criteria

#### 2.2.1. Concept

The focus of this review is how guideline development groups addressed some of the pre-identified challenges when issuing public health guidance. This includes the methods used for identifying different stakeholders and incorporating their perspectives; methods for identification and prioritization of any non-health outcomes; methods for determining thresholds for decision-making; methods for incorporating and grading evidence from non-randomized studies; methods for addressing concerns with conditional recommendations in public health; and methods for reaching consensus and the formal approval of recommendations.

#### 2.2.2. Context

We will include only public health guidelines that used the GRADE approach made from a population perspective, i.e., not intended to cover questions about preventive or treatment measures in any specific group of individuals. Guidelines can be targeting policy makers and cover questions of health policies, management, and broader public policies. We will include only the most recent version of the guideline (the latest update). We will include guidelines published in any language. Geographically, guidelines of all scopes can be included: local, national, regional, or global.

#### 2.2.3. Types of Sources

This review will focus on guidelines for public health interventions related to health protection, health services, and health improvement, dealing with mostly primordial and primary prevention. It will mainly include interventions implemented on a population level, e.g., population-level prevention programs, health system reform, regulation of unhealthy commodities, infrastructure development, social security policies, and the reduction of health inequalities. To state a few examples: screening/prophylaxis, infection prevention and control, eradication efforts, vaccination and related topics, healthcare management, healthcare services, environmental health, promotion of health in populations outside of the healthcare system (e.g., schools). Secondary and tertiary prevention guidelines covering topics such as the prevention of specific conditions in a healthcare setting, prevention of fractures in long-term facilities, prevention of surgical site infections, and the prescription of preventive medications (e.g., statins) will be excluded as these often do not use the population perspective and are linked to the clinical setting. We assume we will not include rapid guidelines as they often do not use robust and transparent methods.

### 2.3. Search

The search will aim to locate public health guidelines that used GRADE. Based on a preliminary search, the pilot search, and consultations with information specialists, we decided to focus on databases and repositories of GRADE (or mostly GRADE) guidelines. The following databases, repositories, and websites will be searched: the GIN international guideline library and registry of guidelines in development (https://guidelines.ebmportal.com/, accessed on 19 September 2021), BIGG international database of GRADE guidelines (https://sites.bvsalud.org/bigg/en/biblio/, accessed on 19 September 2021), Epistemonikos GRADE guidelines repository (https://www.epistemonikos.org/en/groups/grade_guideline, accessed on 19 September 2021), GRADEpro Database of GRADE EtD’s and Guidelines (https://gradepro.org/guidelines/, accessed on 19 September 2021), MAGICapp (https://app.magicapp.org/#/guidelines, accessed on 19 September 2021), National Institute for Health and Care Excellence (NICE) website (https://www.nice.org.uk/guidance/published?type=ph, accessed on 19 September 2021), and the World Health Organization website (https://www.who.int/publications/i, accessed on 19 September 2021). The search will be mostly manual and will not use any text, keywords, or index terms. The reference list of included guidelines will not be searched as it is unlikely that this would result in additional relevant guidelines; however, the citations recommended by guideline authors (either on the websites or in the introductory parts of the documents) will be screened for relevant documents. If the identified document is found to be part of a bigger set of guidelines (e.g., Module 1, Module 2) or a Supplementary Document, all of the related documents and any Supplementary Files or tools will be collated in one folder and regarded as one guideline. Any related documents found on the websites/source of the citation or in the reference lists will be collated and used during extraction (e.g., stakeholder workshops, minutes, recordings, methodological manuals, annexes). Only the latest version of the guideline will be used unless previous versions are referenced as providing necessary information for the interpretation of the updated version. Guidelines published in any language will be included. We will limit the search to 2013–2021 because we are looking for recent developments in the field. The year 2013 has been agreed on by an expert group due to major developments in using GRADE in public health occurring around that time [11,12,13]. The guideline authors will not be contacted; only information provided in the guidelines will be used.

### 2.4. Evidence Selection

During the manual search and title screening, the senior researchers and information specialists will note the potentially relevant records that have been identified into a Microsoft Excel sheet (title, organization/author, publication date, source, URL) and manually remove duplicates. Two reviewers will independently screen full texts of the identified documents against the eligibility criteria. Any disagreements will be resolved via discussion or with a third reviewer. A simple screening algorithm will be used (see Table S1). The relevant full texts of guidelines will be collated and uploaded into an online shared folder, including any related documents and Supplementary Material. The results of the search and the study inclusion process will be reported in full in the final scoping review and presented in a Preferred Reporting Items for Systematic Reviews and Meta-analyses (PRISMA) flow diagram [10].

### 2.5. Data Extraction

Data will be extracted from guidelines included in the scoping review by a team of experienced reviewers who will hold regular meetings for consultations on any issues that may arise. Furthermore, the meetings will aim to ensure that all reviewers are trained and consistent. The project leader will revise the data extracted for each guideline. The following data will be extracted:

- Title of the document;
- Date of publication;
- Source;
- The developing organization or author´s names;
- Characteristics of the guideline (de novo, adopted, living, an update, etc.);
- Purpose of the guideline, including a remit of the organization;
- Target audience;
- Country of origin (if applicable);
- Methods used for identifying different stakeholders and incorporating their perspectives;
- Methods for identification and prioritization of any non-health outcomes;
- Methods for determining thresholds for decision-making;
- Methods for incorporating and grading evidence from non-randomized studies;
- Methods for addressing concerns with conditional recommendations in public health;
- Methods for reaching consensus and the formal approval of recommendations;
- Additional methodological concerns and/or challenges noted by guideline authors;
- Any modifications made to GRADE and their justifications.

A draft extraction form is provided in Table S2 in the Supplementary Materials. Data on the methods used in the respective guidelines will be extracted in the form of: (a) reproduction of the relevant text; (b) yes/no responses; (c) if none of the above is possible, interpretation may sometimes be needed (e.g., interpretation of the evidence profiles). The last option will always be checked by another extractor. The questions and items of the data extraction form were revised based on the piloting phase and consultations with GRADE methodologists.

### 2.6. Data Analysis and Presentation

A combination of content analysis (deductive and inductive) and descriptive statistics will be used for data analysis. Some of the collected data (the yes/no items) will be quantified, and simple descriptive statistics (distribution) will be used for analysis.

To analyze the textual data, we will use directed content analysis [14], imposing predefined evidence/theory from the recent GRADE Public Health Group paper [5] on the data while also allowing emergent codes to be identified. The paper describes five challenges when using the GRADE approach to develop public health guidelines: (1) incorporating diverse perspectives, (2) selecting and prioritizing outcomes, (3) interpreting outcomes and identifying a threshold for decision-making, (4) assessing the certainty of evidence from diverse sources, including non-randomized controlled studies (NRS), and (5) addressing implications for decision-makers, including concerns about conditional recommendations. These challenges will each constitute one theme. Based on other recent work of the GRADE Working Group, we will elaborate on some of the challenges (e.g., for theme 5, we will also explore the use of good practice statements and their equivalents) and add one more theme: (6) formulating and agreeing on recommendations. Furthermore, we will address any new challenges and any modifications made to GRADE. If we identify additional challenges besides those mentioned above, we will add new themes. We will use a combination of deductive and inductive content analysis.

Two coders will independently read the data extracted from the guidelines twice. During the coding process, the coders will highlight the important and relevant portions of the text and choose a word, phrase, or description to represent the meaning of the text segment. The codes that were developed in the pilot phase will be used as predefined codes in the full analysis, and new ones will be added. Codes with similar concepts will be grouped together to form categories. Where appropriate, codes or categories will be accompanied by explanations and examples from the text. Coders will not aim to quantify the codes’ occurrence. We will use the codes and categories to populate the six predefined themes, and add new themes as needed.

In the final review, we will present the results of the search in a modified PRISMA flowchart [10], the summary of the basic characteristics of the identified guidelines, and the results of the screening process. Analysis results will be presented narratively, codes and categories for each theme, where applicable, will be presented in a tabular form, and the quantified data will be presented in graphs. Any deviations and changes from the protocol will be reported in the full review.

The whole process was piloted on public health guidelines published from January to August 2021; the results are presented below. The purpose of the pilot phase was to: (a) test and improve the methods proposed in the protocol; (b) develop and improve the necessary tools; (c) refine the process and content of data extraction; (d) assemble and train a team of reviewers and set up the necessary group processes; and (e) determine the scope of the work. We used a modified approach for the pilot, and after its completion, refined the methods of the proposed scoping review.

## 3. Preliminary Results (Pilot Phase) and Discussion

In August 2021, we manually searched the databases and repositories according to the protocol outlined above for any relevant public health guidelines that used the GRADE approach and were issued between January and August 2021, all languages included. Two independent reviewers screened the full texts of documents against the screening criteria and identified 13 relevant guidelines. One was issued by the Registered Nurses’ Association of Ontario (RNAO, Boucherville, QC, Canada), one by the National Institute for Health and Care Excellence (NICE, London, UK), and the remaining 11 by the World Health Organization (WHO) (Table 1).

One reviewer extracted relevant text and data from the guidelines and performed the analysis as specified in the protocol above, and two other co-authors reviewed the work. The results of this pilot analysis of the 13 public health guidelines are organized around the six predefined themes. No conclusions should be drawn based on the pilot analysis, as its purpose was to determine the best approach to conducting the full review. Therefore, we do not provide a discussion of the pilot results here.

For theme 1 (incorporating diverse perspectives and identifying stakeholders), we extracted relevant text related to the methods for identifying stakeholders and including them in the guideline development process and performed a content analysis. The results are shown in Table 2.

**Figure 1:**
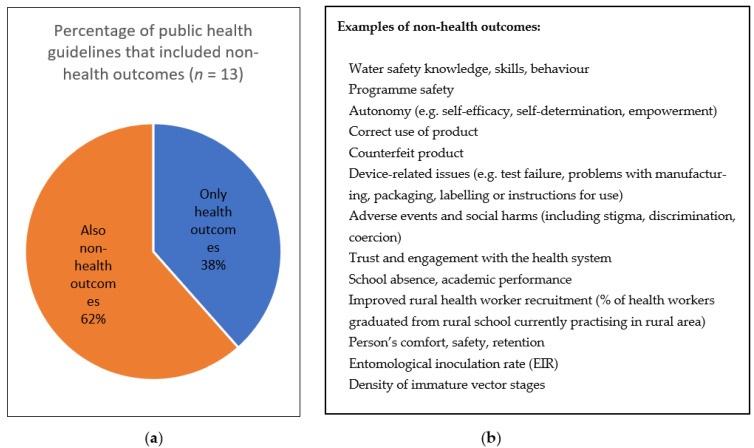
(**a**) Percentage of public health guidelines (n = 13) published in January–August 2021 that included non-health outcomes; (**b**) examples of non-health outcomes in the guidelines.

**Figure 2:**
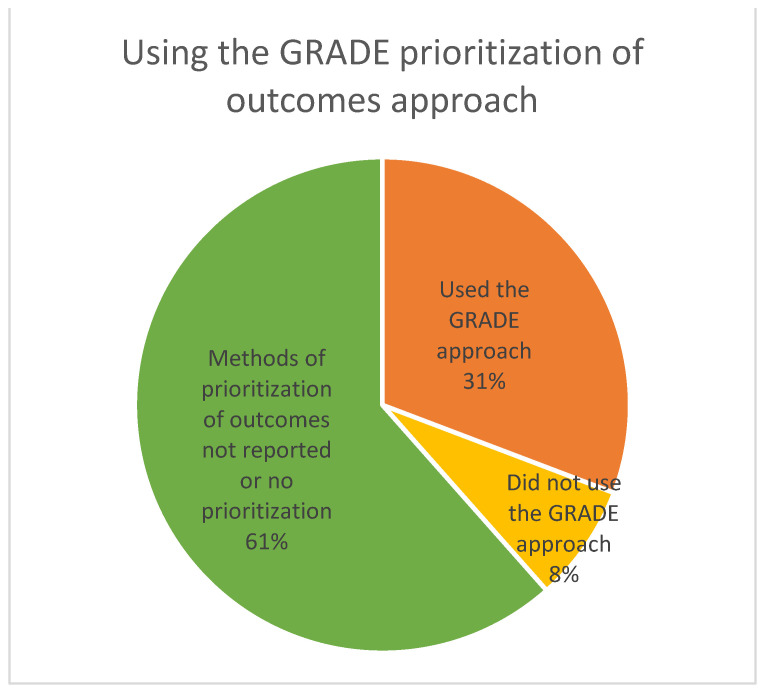
The use of the GRADE approach to prioritization of outcomes. (The GRADE approach defined as: ranking of outcomes 1–9, and dividing into critical, important, non-critical).

For theme 2 (identifying and prioritizing outcomes), we extracted the number of guidelines that used non-health outcomes and provided examples (Figure 1) and the number of guidelines that used the GRADE approach for prioritization of outcomes (Figure 2). For content analysis in theme 2, we extracted and coded text related to how outcomes were identified and selected, and what methods, if any, were used for their prioritization (Table 3). In the full review, we will also present the non-GRADE approaches to outcome prioritization and any modifications to the GRADE approach.

Due to a lack of data, we did not analyze the data for theme 3 (interpreting outcomes and identifying a threshold for decision-making) in the pilot phase. No data has been found in the guidelines to populate theme 3.

**Figure 3:**
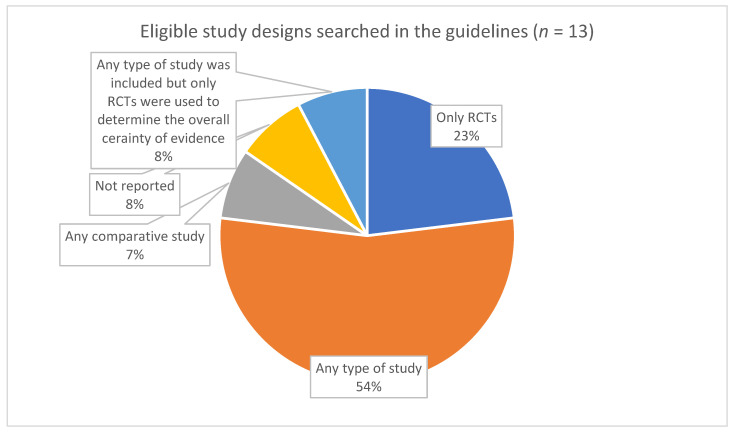
Study designs used to inform the guidelines.

**Figure 4:**
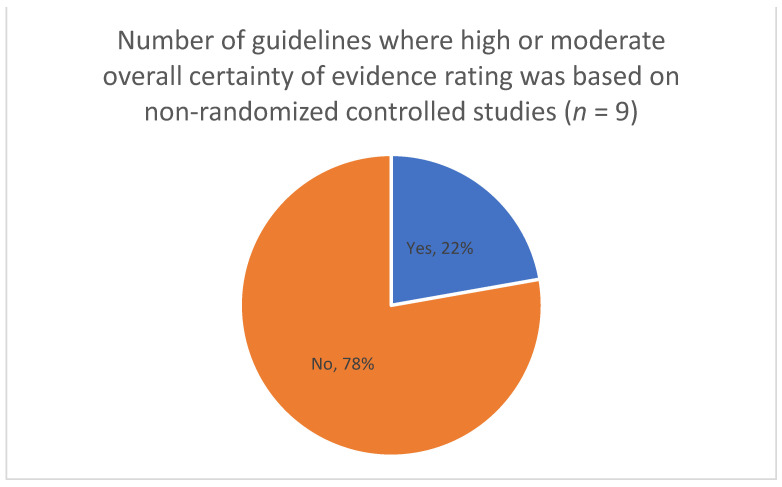
Moderate or high certainty of evidence rating based on non-randomized studies.

**Figure 5:**
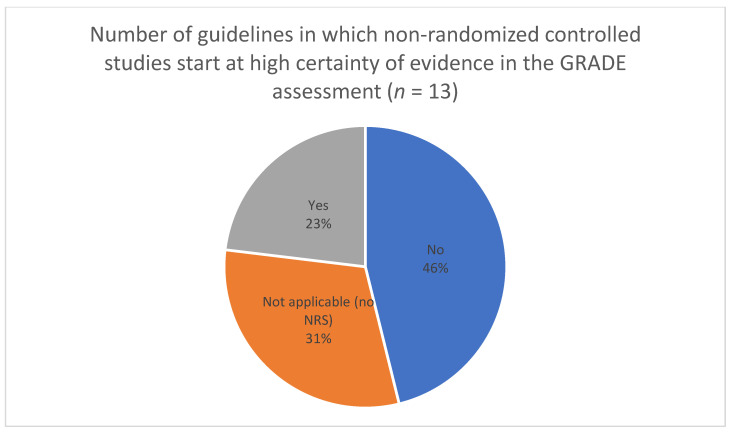
Certainty of evidence assessment for NRS—initial rating at high.

**Figure 6:**
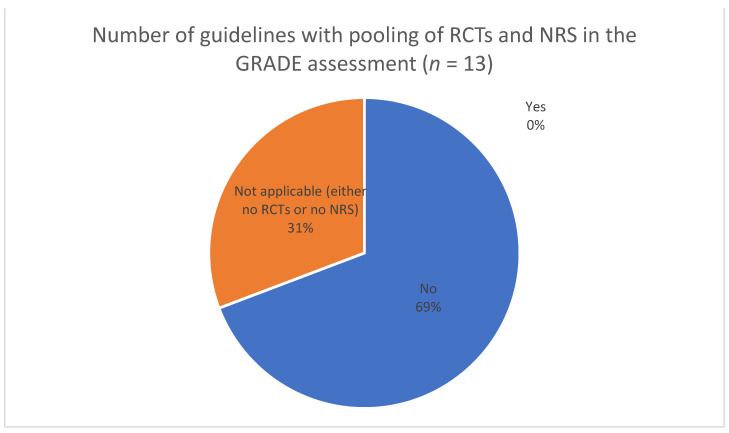
Pooling of RCTs and NRS when assessing the certainty of evidence.

For theme 4 (assessing certainty of evidence from diverse sources, including NRS), we extracted information on which study designs were used to inform the guidelines (Figure 3), whether evidence from NRS led to a ranking of moderate or high certainty of evidence (CoE) (Figure 4), the number of guidelines in which NRS started with high CoE (Figure 5), and whether RCTs and NRS evidence was pooled when assessing CoE (Figure 6). In the full review, these data will be accompanied by an analysis of the specific reasons and/or examples of assessing moderate or high CoE based on NRS evidence and of the rationale for NRS starting at high CoE and for pooling of RCTs and NRS. Furthermore, we will be extracting and analyzing data on the methods for assessing the overall CoE— whether this was done in the guideline or whether the GRADE approach was used—and providing a description of any modifications to GRADE in assessing the overall CoE.

**Figure 7:**
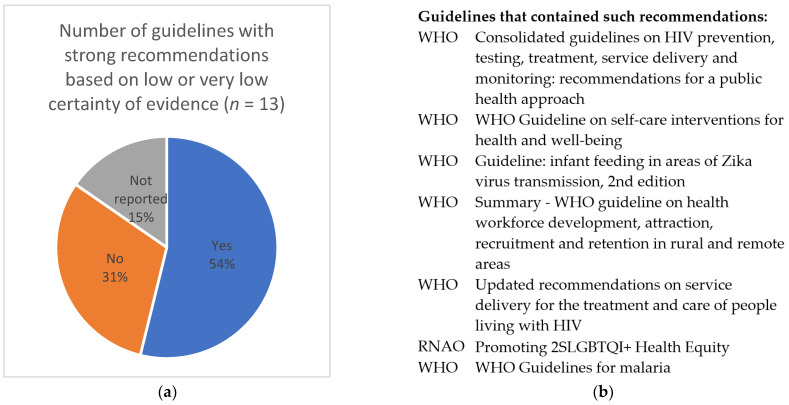
(**a**) Developing strong recommendations based on low or very low certainty of evidence; (**b**) a list of guidelines with strong recommendations based on low or very low certainty of evidence.

For theme 5 (addressing implications for decision-makers, including concerns about conditional recommendation), we first identified whether the guideline included strong recommendations based on low or very low CoE (Figure 7). The units of analysis were the extracted texts on the rationale for such recommendations. We identified the codes and developed categories describing the panels’ different reasons for developing strong recommendations based on low or very low CoE. We then added further explanations and examples of such recommendations, one for each category (Table 4). Four reasons have been previously identified by Hilton Boon et al. [5]: life-threatening situations; uncertain benefit but certain harm; potential equivalence of effectiveness in which one option is clearly more or less risky or costly; and potential for catastrophic harm. We will include these as predefined categories in the full review. Here, we only present the newly identified categories (Table 4).

For theme 6 (formulating and agreeing on recommendations), the unit of analysis were texts related to the methods for reaching consensus and agreeing on recommendations. We identified text relevant to the course of the guideline development group (or panel) meetings for formulating and agreeing on recommendations, how the panel prepared for the meeting (what happened prior to the meeting), how those meetings were facilitated and by whom, how the panel agreed on recommendations, and the specific voting thresholds if voting was used (Table 5).

### Limitations and Strengths

The work presented here serves as a protocol for a scoping review. This work does not present the results of primary research. The aim of protocols is, generally, to provide a detailed outline of the proposed review project [7]. The review itself should not start before the protocol is finalized, and ideally, published. We have rigorously followed the JBI methodology for scoping reviews and protocols and included all necessary information in detail [7]. Furthermore, we have piloted the review methods to make sure all of the steps are feasible to carry out. We expect the work to be very extensive, and the review, therefore, is the result of many international experts in the field working in cooperation.

This work should be viewed, first and foremost, as a protocol for a scoping review. The reporting of the preliminary pilot results serves solely to explain the proposed methods better, especially how the analysis will be done and in what way the full results will be reported. The main limitation of this paper is, therefore, that it does not include the full results, discussion, and conclusion.

From the pilot results, it can already be seen that most of the identified guidelines were issued by the World Health Organization (WHO). It brings forward the question of over-saturation of the same kind of data if the WHO has used more or less the same methods. So far, we have observed that, although the WHO methods are well described, there are modifications to the usual processes in many of the public health guidelines that have been issued. We are confident it is worthwhile to include large numbers of guidelines even when they are issued by the same organization. In the preliminary full search in years 2013–2021, however, there were many guidelines issued by organizations other than WHO and it seems that the full review will comprise a much wider variety of issuing organizations then was the case in the pilot phase. The limited number of issuing organizations may have been caused by the COVID-19 pandemic.

Lastly, we have not included any COVID-19 guidelines in the pilot phase as none fulfilled the eligibility criteria of this review (e.g., excluded for one or more of the following reasons: rapid guideline methodology; mostly focusing on treatment or individual perspective rather than public health perspective; not using the GRADE approach; not clearly defined as a guideline; methods not described appropriately).

## 4. Conclusions

This protocol provides a description of the objectives, inclusion criteria, methods, and analysis of a scoping review to be undertaken by an international group of experts, building on the work of the GRADE Public Health Group in addressing challenges in public health guideline development. It concludes with the pilot phase results, during which 13 public health guidelines issued between January and August 2021 were analyzed. We draw no conclusions from this limited pilot evidence. The piloting phase was conducted to refine the proposed methods and to further develop the necessary tools (e.g., data extraction tool). After the piloting phase, the objectives have been rephrased more clearly, the eligibility criteria refined, the simple screening tool tested, and the data extraction tool modified and placed in an online form. The basic outline of the workflow and communication methods between team members had been established. The pilot analysis helped to outline the predefined themes and identify new categories to be used in the full review. We will publish the full results of the scoping review in a peer-reviewed journal when available. The full review will aim to provide concrete examples of how GRADE is currently being used in public health guidelines, as well as a discussion on which parts of the current GRADE guidance need to be adapted to be better suited for public health topics. The results of the review will be used to further address the challenges of using GRADE in the development of public health guidelines and, together with other research, will form a basis for GRADE concept articles or guidance on the topic. The work will benefit all relevant stakeholders (public health policy makers, governmental and non-governmental organizations, professional organizations, and all end-users of public health interventions) involved in the development, dissemination and implementation of public health guidelines.

## Figures and Tables

**Table 1:** A list of identified guidelines and their basic characteristics.

Title and Reference	Publication Date	Organization	Purpose
WHO antenatal care recommendations for a positive pregnancy experience: nutritional interventions update: zinc supplements during pregnancy [15]	12 August 2021	WHO	To reflect and respond to issues surrounding antenatal care To prioritize person-centred health and well-being
WHO Guideline on the prevention of drowning through provision of day-care and basic swimming and water safety skills [16]	23 July 2021	WHO	To provide recommendations about appropriateness of day-care and basic swim skills and water safety among children (low- and middle-income countries)
Consolidated guidelines on HIV prevention, testing, treatment, service delivery and monitoring: recommendations for a public health approach [17]	16 July 2021	WHO	To provide recommendations about providing ARV drugs for HIV prevention and treatment (settings with limited health system capacity and resources); to increase HIV prevention, testing, and treatment access, strengthen the HIV care and integrate the provision of ARV drugs; to provide guidance on adapting, setting priorities for and implementing the clinical and operational recommendations, and monitoring their implementation and impact.
Recommendations and guidance on hepatitis C virus self-testing [18]	15 July 2021	WHO	To supplement the existing WHO guidelines on hepatitis testing services; to support countries and national programmes in reaching 2030 HCV elimination goals by helping them reach people who may not otherwise test.
WHO Guideline on self-care interventions for health and well-being [19]	13 July 2021	WHO	To provide guidance, to support individuals, communities, and countries with quality health services and self-care interventions good practice statements on key issues, to promote and increase safe and equitable access and the uptake and use of self-care interventions for health considerations for future research and guidelines processes.
WHO guideline on school health services [20]	22 June 2021	WHO	To provide guidance on effectiveness, acceptability, and content of SHS involving a health worker; to support national governments and partners to develop SHS programmes.
Guideline: infant feeding in areas of Zika virus transmission, 2nd edition [21]	15 June 2021	WHO	To provide recommendations on infant feeding in areas with Zika virus transmission.
WHO guideline on health workforce development, attraction, recruitment and retention in rural and remote areas [22]	6 May 2021	WHO	To support national authorities in strengthening the density and capacity of health workforce (rural and remote areas); to develop, attract, recruit, and retrain health workers; to identify relevant elements and implementation and evaluation considerations at policy and system levels.
Updated recommendations on service delivery for the treatment and care of people living with HIV [23]	28 April 2021	WHO	To encourage improvement in access to ART; to simplify care delivery; to support return to care for those who have disengaged; to support reduction of people acquiring HIV and dying for associated causes; to contribute to achieving the Triple-Billion targets.
WHO guideline on the dairy protein content in ready-to-use therapeutic foods (RUTF) for treatment of uncomplicated severe acute malnutrition [24]	8 April 2021	WHO	To provide recommendation on whether reduced dairy or non-dairy RUTF should be used for treating malnutrition.
Promoting 2SLGBTQI+ Health Equity [25]	July 2021	RNAO (CA)	To provide recommendations on care practices for 2SLGBTQI+ people; to enhance safety of organizations for 2SLGBTQI+ people; to optimize health outcomes for those people.
Behaviour change: digital and mobile health interventions [26]	7 October 2020 ^1^	NICE (UK)	To cover interventions that use digital or mobile platforms to help people change behaviour.
WHO Guidelines for malaria [27]	13 July 2021	WHO	To reduce and ultimately eliminate malaria; to provide recommendations for malaria prevention; to support the development national malaria policies for prevention; to maximize the impact of available resources.

WHO—World Health Organization, NICE—National Institute for Health and Care Excellence, RNAO—Registered Nurses’Association of Ontario, CA—Canada, UK—United Kingdom. ^1^ We included this guideline to incorporate more organizations in the pilot extraction.

**Table 2:** Categories and codes for theme 1: incorporating diverse perspectives and identifying stakeholders.

THEME 1 (Predefined): Incorporating Diverse Perspectives and Identifying Stakeholders	
CATEGORIES	CODES
Methods for identifying members of GDG	CODE: identifying members by searching the literature for researchers in the topic area CODE: identifying members through key informant interviews CODE: identifying members through established professional networks, associations, organizations, and advocacy bodies CODE: suggestions for membership came from previous guideline development group memberships CODE: identifying members in accordance with the organization´s Guideline development manual CODE: suggestions for membership came from the Guideline endorsing organization and its dedicated departments, expert advisory panels, country and regional offices and groups CODE: a group appointed by the endorsing organization identified experts for GDG
Characteristics of GDG members	CODE: GDG includes commissioners CODE: GDG includes health economist CODE: GDG includes local government CODE: members have expertise in guideline development methods CODE: members have collectively the necessary multidisciplinary expertise in the topic of interest CODE: members are among the intended end-users CODE: members have expertise in research CODE: members are representatives of civil society organizations CODE: members are from the target population (networks, lay members) CODE: representatives from national programmes to provide perspectives on the resource implications in their countries CODE: absence of significant conflicts of interest CODE: external (outside of the endorsing organization) CODE: geographically dispersed CODE: gender-balanced
Procedures of forming GDG	CODE: GDG membership list was posted for public review and comment and then finalized

GDG—guideline development group.

**Table 3:** Categories and codes for theme 2.

THEME 2 (Predefined): Identifying and Prioritizing Outcomes	
CATEGORIES	CODES
Identifying and selecting outcomes	CODE: due to the variability of outcome reporting, decision rules for selecting outcomes were used CODE: outcomes identified via review of the literature CODE: outcomes identified via key informant interviews and discussion groups CODE: outcomes identified via expert panel survey prior to the in-person meeting CODE: outcomes identified via expert panel discussion at an in-person meeting CODE: primary outcomes were agreed upon by the GDG [seems after the relevant studies were identified, not a priori] CODE: scoping exercise of guidelines and systematic reviews of the Guideline topics informed the outcomes CODE: scoping review of target population´s values and preferences informed the outcomes
Prioritizing outcomes	CODE: outcomes were aligned with the Sustainable Development Goals CODE: priority outcomes were aligned with a previous (related) guideline CODE: Critical and important outcomes were agreed between the review team and the Steering Group and were endorsed by the GDG CODE: outcomes were prioritized via an online survey–members ranked the importance of each outcome on the GRADE rating scale of 1–9 (0–3: not important; 4–6: important; 7–9: critical). CODE: up to five priority outcomes were determined based on confidential voting by each member, and a subsequent facilitated discussion of the voting results CODE: online vote determined critical outcomes if 70% of the votes were ranked 7–9 on a 9-point Likert scale

GDG—guideline development group.

**Table 4:** Categories for theme 5 with example recommendations.

THEME 5: Addressing Implications for Decision-Makers, Including Concerns about Conditional Recommendations Reasons for Developing Strong Recommendations Based on Low or Very Low Certainty of Evidence		
Category	Explanation (by the Review Team)	Example Recommendation
There is a substantial experience of using the intervention (already widely implemented) and no harm.	The “intervention” is already implemented, seems effective, and this is causing the lack of research leading to low or very low certainty of evidence.	*“WHO recommends making the self-management of folic acid supplements available as an additional option to health worker-led provision of folic acid supplements for individuals who are planning pregnancy within the next three months.”* [19]
Greatly valued and/or needed by the target population and no known harm.	The target population is suffering greatly from a problem (or an intervention is needed), and any non-harmful intervention will be greatly valued and likely effective. Assessing effectiveness/costs and other aspects seems secondary.	*“WHO recommends investing in rural infrastructure and services to ensure decent living conditions for health workers and their families.”* [22]
Using other types of evidence with high confidence (indirect, pharmacokinetic modelling, programmatic data).	Various other-than scientific data (not experience or expert evidence) are available, and the panel has high confidence in them, or is confident that the identified indirect evidence can completely substitute the missing direct evidence (e.g., when one disease has much more evidence than another, but they are essentially the same, common for infectious diseases).	*“Children weighing < 20 kg should receive a higher dose of artesunate (3 mg/kg bw per dose) than larger children and adults (2.4 mg/kg bw per dose) to ensure equivalent exposure to the drug.”* (artesunate is recommended for adult populations with high certainty of the evidence) [27]
Potentially equivalent in benefits and harms, and doing intervention (vs. not doing) seems better in all other EtD domains (no reasons against).	When considering whether to perform an intervention or not, in the context of no obvious effects or harms, one option seems better in all other aspects, and there seems to be no reason not to perform the intervention.	*“WHO recommends designing and enabling access to continuing education and professional development programmes that meet the needs of rural health workers to support their retention in rural areas.“* [22]
The intervention is ethically necessary (“sound”, “basic human right”).		*“WHO recommends ensuring a safe and secure working environment for health workers in rural and remote areas.”* [22]
A perfect balance of effects, the only problem is low or very low certainty of evidence (lack of higher-certainty research)	Recommendation formulated in the context of lack of higher-certainty evidence (usually due to limited evidence, e.g., only observational studies downgraded by 0 or 1 level, or RCTs downgraded by 2–3 levels) when all other aspects are in favour of the intervention.	*“People established on anti-retroviral therapy should be offered refills lasting 3–6 months, preferably six months if feasible.”* [23] Rationale (from the guideline): “Some of the evidence supporting these recommendations came from observational studies with methodological limitations, and there was important variability (heterogeneity) in outcomes across studies.”

**Table 5:** Categories and codes for theme 6.

THEME 6 (Predefined): Formulating and Agreeing on Recommendations	
CATEGORIES	CODES
Prior to the meeting for agreeing on recommendations	CODE: PanelVoice was used to allow panelists to pre-vote on the EtD framework questions CODE: members could draw attention to any new evidence prior to the meeting CODE: recommendations were drafted a priori to the meeting CODE: members received detailed background documents prior to meetings CODE: reviewed the preliminary judgements and comments posted by all members in the online EtD form CODE: reviewed evidence summaries CODE: all members posted comments in an online form (EtD) CODE: members were provided with the EtD frameworks, evidence profiles, and full-text articles
The course of the meetings to agree on recommendations	CODE: discussion facilitated by the chair and/or methodologist CODE: the meeting was guided by a clear protocol CODE: process guided by the organization´s manual/handbook CODE: detailed background documents had been summarized in presentations during each GDG meeting CODE: methodologist facilitated discussions CODE: discussion was facilitated by co-chairs and methodologists CODE: formulate recommendations through a process of group discussion, engagement, and revision CODE: members were presented with a ‘neutral’ recommendation and decided on its direction and strength
Facilitation methods	CODE: voting was used as a starting point to build consensus (not as a formal vote) CODE: members were asked to raise their hands in support of each separate option as a decision-making aid (not as a formal vote) CODE: in the online environment, close attention was paid to eliciting responses from all members CODE: regular straw-polling and chat function were used to gain an initial indication of members’ views
Methods for agreeing on recommendations	CODE: consensus—unanimous agreement (on the direction, strength, and wording of a recommendation) after a facilitated discussion CODE: voting only in case of disagreement CODE: forming recommendations without explicitly considering those from a previous version of the guideline CODE: no member expressed opposition to the recommendations CODE: at an in-person meeting CODE: at an online meeting CODE: online vote (after the meeting) CODE: if consensus could not be reached, more time was given for deliberations CODE: the GDG reached an agreement (method not specified) CODE: voting was anonymous (“In some cases, anonymous voting was used for judging the different criteria and developing the final recommendation to reduce peer pressure.”) CODE: every member was asked to express their decision verbally
Voting thresholds	CODE: voting, 60% of the cast votes CODE: voting, 70% of the cast votes CODE: voting, two thirds of the cast votes

GDG—guideline development group, EtD—evidence-to-decision.

## Data Availability

All data is provided in the article.

## Supplementary Materials

The following are available online at https://www.mdpi.com/article/10.3390/ijerph19020992/s1, Table S1: Screening questions, Table S2: Data extraction instrument.

## References

[1] World Health Organization (2003). Guidelines for WHO Guidelines.

[2] Qaseem A., Forland F., Macbeth F., Ollenschläger G., Phillips S., van der Wees P. (2012). Guidelines International Network: Toward international standards for clinical practice guidelines. Ann. Intern. Med..

[3] Schünemann H., Brożek J., Guyatt G., Oxman A. (2013). GRADE Handbook.

[4] Norris S.L., Rehfuess E.A., Smith H., Tunçalp Ö., Grimshaw J.M., Ford N.P., Portela A. (2019). Complex health interventions in complex systems: Improving the process and methods for evidence-informed health decisions. BMJ Glob. Health.

[5] Hilton Boon M., Thomson H. (2021). Challenges in applying the GRADE approach in public health guidelines and systematic reviews: A concept article from the GRADE Public Health Group. J. Clin. Epidemiol..

[6] Munn Z., Peters M.D.J. (2018). Systematic review or scoping review? Guidance for authors when choosing between a systematic or scoping review approach. BMC Med. Res. Methodol..

[7] Peters M.D.J., Godfrey C., Aromataris E., Munn Z. (2020). Chapter 11: Scoping Reviews (2020 Version). JBI Manual for Evidence Synthesis.

[8] Peters M.D.J., Marnie C. (2020). Updated methodological guidance for the conduct of scoping reviews. JBI Evid. Synth..

[9] Tricco A.C., Lillie E. (2018). PRISMA Extension for Scoping Reviews (PRISMA-ScR): Checklist and Explanation. Ann. Intern. Med..

[10] Page M.J., McKenzie J.E. (2021). The PRISMA 2020 statement: An updated guideline for reporting systematic reviews. J. Clin. Epidemiol..

[11] Akl E.A., Kennedy C. (2012). Using GRADE methodology for the development of public health guidelines for the prevention and treatment of HIV and other STIs among men who have sex with men and transgender people. BMC Public Health.

[12] Rehfuess E.A., Akl E.A. (2013). Current experience with applying the GRADE approach to public health interventions: An empirical study. BMC Public Health.

[13] Burford B.J., Rehfuess E. (2012). Assessing evidence in public health: The added value of GRADE. J. Public Health.

[14] Hsieh H.-F., Shannon S.E. (2005). Three Approaches to Qualitative Content Analysis. Qual. Health Res..

[15] World Health Organization (2021). WHO Antenatal Care Recommendations for a Positive Pregnancy Experience: Nutritional Interventions Update: Zinc Supplements during Pregnancy.

[16] World Health Organization (2021). WHO guideline on the Prevention of Drowning through Provision of Day-Care and Basic Swimming and Water Safety Skills.

[17] World Health Organization (2021). Consolidated Guidelines on HIV Prevention, Testing, Treatment, Service Delivery and Monitoring: Recommendations for a Public Health Approach.

[18] World Health Organization (2021). Recommendations and Guidance on Hepatitis C Virus Self-Testing.

[19] World Health Organization (2021). WHO Guideline on Self-Care Interventions for Health and Well-Being.

[20] World Health Organization (2021). WHO Guideline on School Health Services.

[21] World Health Organization (2021). Guideline: Infant Feeding in Areas of Zika Virus Transmission.

[22] World Health Organization (2021). WHO Guideline on Health Workforce Development, Attraction, Recruitment and Retention in Rural and Remote Areas.

[23] World Health Organization (2021). Updated Recommendations on Service Delivery for the Treatment and Care of People Living with HIV.

[24] World Health Organization (2021). WHO Guideline on the Dairy Protein Content in Ready-to-Use Therapeutic Foods for Treatment of Uncomplicated Severe Acute Malnutrition.

[25] Promoting 2SLGBTQI+ Health Equity (Registered Nurses’ Association of Ontario). https://rnao.ca/bpg/guidelines/promoting-2slgbtqi-health-equity.

[26] Behaviour Change: Digital and Mobile Health Interventions (National Institute for Heatlh and Care Excellence (NICE)). www.nice.org.uk/guidance/ng183.

[27] World Health Organization (2021). WHO Guidelines for Malaria.

