# Supplementary material for: Addressing Challenges When Applying GRADE to Public Health Guidelines: A Scoping Review Protocol and Pilot Analysis
Source: Int J Environ Res Public Health. 2022 Jan 16;19(2):992. doi: 10.3390/ijerph19020992 (PMC8775462; doi:10.3390/ijerph19020992)
Supplement: Supplementary file 1 [file ijerph-19-00992-s001.zip › ijerph-1484828-SI.pdf]

# Supplementary Material

## Table S1: Screening questions

| Is the document a guideline? | Did the guideline use the GRADE approach for developing recommendations? | Did the guideline provide evidence-to-decision (EtD) tables or an equivalent (e.g. all EtD sections covered, but only provided in a narrative way in the text of the guideline)? | Is the guideline an original main document? (e.g. it is not an annex, it is not one part of a range of guidelines on the same topic) | Is the guideline a public health guideline (see protocol for detailed definition)? | Does the guideline cover screening topics? (At least 50% of recommendations are related to screening.) |
|---|---|---|---|---|---|
| Yes – include<br>No - exclude | Yes – include<br>No - exclude | Yes – include<br>No - exclude | Yes – include<br>No – look for the main document | Yes – include<br>No - exclude | Yes – include as a screening guideline<br>No – include |

## Table S2: Data extraction instrument

| |
|---|
| Purpose (scope) of the guideline, including remit of the organisation (if stated) |
| Target audience (who will use the guidelines, who is it for) |
| How were stakeholders for the guideline identified? |
| How were outcomes identified and prioritized? |
| Was the GRADE approach to prioritizing outcomes used? |
| Were there any non-health outcomes? (examples: academic achievement, job satisfaction) |
| How did the panel/guideline development group decide on thresholds for decision-making (or minimal important difference, or important thresholds)? How were findings interpreted? |
| How did the panel/group assess the overall certainty of evidence? |
| What consensus methods were used to agree on the recommendations and their strength? |
| How many recommendations were based on high/moderate/low/very low certainty of evidence respectively? |
| Were there any strong recommendations based on low or very low certainty of evidence? |
| Were there any good practice statements in the guideline (or any formally approved statements other than recommendations)? |
| What study designs were included in the guideline? (examples: only RCTs, any type of study) |
| Were non-randomized controlled studies (NRS) used in the guideline? |
| Was there moderate or high certainty of evidence based on NRS/observational studies? |
| Was evidence from RCTs and NRS/observational studies pooled together in GRADE tables? |
| Did NRS or observational studies start at high certainty of evidence and then downgraded? |
| Were there any methodological concerns and/or challenges noted by guideline authors? |
| Were there any modifications made to the GRADE approach, and how were they justified? |

Abbreviations: RCT – randomized controlled trial; NRS – non-randomized studies of interventions, EtD – evidence-to-decision framework
